# Reframing participation through empowerment: Mechanisms driving Chinese women’s intentions in adventure sports tourism

**DOI:** 10.1371/journal.pone.0339943

**Published:** 2026-05-12

**Authors:** Wenhao Guo, Yuanji Zhong, Yongshun Wang, Pengwei Chen, Shiqi Zhuang, Jingjin Liu

**Affiliations:** 1 School of Recreational Sports and Tourism, Beijing Sport University, Beijing, China; 2 School of Physical Education and Arts, Jiangxi University of Science and Technology, Ganzhou, Jiangxi, China; 3 College of Physical Education, Huaqiao University, Quanzhou, Fujian, China; 4 Department of Health and Physical Education, The Education University of Hong Kong, Hong Kong SAR, China; 5 Department of Geriatrics, Shenzhen People’s Hospital (The First Affiliated Hospital, Southern University of Science and Technology; The Second Clinical Medical College, Jinan University), Shenzhen, Guangdong, China; 6 Guangdong Provincial Clinical Research Center for Geriatrics, Shenzhen Clinical Research Center for Geriatrics, Department of Geriatrics, Shenzhen People’ s Hospital, Shenzhen, Guangdong, China; Sichuan Agricultural University, CHINA

## Abstract

Women’s participation in adventure sports tourism is growing in China but remains uneven under collectivist traditions and gendered expectations. Drawing on Self-Determination Theory and Empowerment Theory, this study examines how intrinsic and extrinsic motivations are associated with participation intentions, mediated by psychological, social, and political empowerment, and moderated by perceived risk. Survey data were collected from 566 women participating in skiing, surfing, and rock climbing at representative destinations. Standardized measures were employed, and moderated mediation analysis with bias-corrected bootstrapping was conducted to assess the hypothesized associations. Results reveal that intrinsic and extrinsic motivations positively predict participation intention via distinct empowerment pathways. While intrinsic motivation acts through psychological and political dimensions, extrinsic motivation engages all three, with political empowerment exerting the strongest mediation effect. Notably, risk perception significantly attenuates these indirect relationships. A key theoretical contribution is the reconceptualization of political empowerment: rather than resistance, it functions as a means of securing institutional legitimacy, proving resilient under elevated risk perception compared to the vulnerability of social empowerment. The study concludes with practical strategies for enhancing political legitimacy and risk management to promote sustained engagement.

## 1. Introduction

Adventure has historically been framed as a masculine pursuit, often associated with physical strength, bravery, and risk-taking [1]. In recent years, however, this association has begun to shift. Adventure is increasingly becoming a space through which women articulate subjectivity and negotiate identity. According to the Travel Trends Report 2025: The Rise of the Female Adventurer, women now account for more than half of global adventure travel bookings, reshaping both the image and the narratives of adventure sports tourism [2]. This development reflects broader changes in travel motivations, which have moved from passive sightseeing toward experiences emphasizing meaning, engagement, and self-affirmation. In this context, adventure sports tourism has grown beyond its niche origins to become a culturally and socially significant practice [3].

In China, these global trends are mirrored but shaped by specific cultural dynamics. Women have traditionally faced structural constraints on participation in physical and high-risk activities, reinforced by institutional barriers and cultural assumptions about gender roles [4]. Discourses rooted in patriarchy often cast women as gentle, restrained, and risk-averse, positioning them at the margins of activities such as climbing, mountaineering, or extreme sports [5]. Such expectations have not only framed women as physically disadvantaged but also narrowed their space for legitimate participation in adventure sports tourism. These boundaries, however, are loosening. Social change and rising gender awareness have encouraged more Chinese women to take up adventure sports once dominated by men. Participation has increasingly been understood not only as physical challenge but also as a form of identity-making and self-assertion [6]. A recent survey by Xinhua News [7] reports that nearly two-thirds of women in urban China now regularly engage in outdoor activities, suggesting greater openness to high-intensity sports and an active redefinition of personal and cultural boundaries. Adventure sports tourism, once a largely male-coded domain, is gradually becoming a cultural site where women seek empowerment and negotiate their place in public space [8]. At the same time, persistent gender hierarchies remain visible, revealing ongoing tensions around social perceptions, access to resources, and risk culture.

Given this landscape, further theoretical and empirical research is needed to clarify how women in China navigate empowerment, body politics, and spatial legitimacy in adventure sports tourism. This study responds by integrating self-determination theory and empowerment theory into a “motivation–empowerment–intention” framework. It examines how intrinsic and extrinsic motivations are associated with participation intention through empowerment, and how risk perception shapes these associations. By focusing on the psychosocial processes underlying Chinese women’s engagement in high-risk adventure contexts, the study seeks to contribute to debates on gender, identity, and leisure, while offering insights for building more equitable participation environments in this growing sector.

### 1.1 Adventure sports tourism

Adventure sports tourism represents a specialized evolution within the broader landscape of adventure tourism. While the latter encompasses a vast array of experiences ranging from cultural expeditions and environmental exploration to high-adrenaline activities [9], adventure sports tourism carves out a distinct niche defined by physical rigor and the deliberate navigation of risk. The demarcation lies in the centrality of sport, as unlike general adventure travel, these pursuits demand active physical engagement and technical proficiency. Activities such as rock climbing, skiing, and surfing are not merely passive observations of nature but require athletic conditioning and psychological resilience [10]. Consequently, the primary motivators shift from general novelty to self-transcendence and the pursuit of intense sensory stimulation [11].

Although the boundaries between these domains are porous, their structural attributes diverge significantly. Adventure tourism is often more heterogeneous and exploratory, whereas adventure sports tourism prioritizes structured and skill-dependent interactions with the environment that are often mediated by standardized safety protocols [12]. Within the typology of adventure activities, this study specifically conceptualizes adventure sports tourism as adventure tourism with sport participation as the primary purpose. This category is characterized by three pillars: a natural or semi-natural setting, a requirement for specialized equipment and physical capabilities, and the presence of identifiable yet manageable risk. This definition synthesizes prior theoretical frameworks that emphasize the athletic and challenge-oriented nature of such engagements [13,14].

### 1.2 Adventure motivation and participation intention

Self-determination theory conceptualizes motivation along a continuum from externally regulated forms to fully internalized, intrinsic motivation, with increasing autonomy in action [15,16]. It distinguishes intrinsic motivation, extrinsic motivation, and amotivation, with the first two shaping behavior in different ways across social settings [17]. In adventure sports tourism, participation generally presumes voluntary choice and goal-directed engagement. The present study therefore concentrates on intrinsic and extrinsic motivation and does not model amotivation. For women, intentions to participate typically emerge from a blend of internal drivers, such as the pursuit of stimulation and the satisfaction of mastering challenges, and external influences, including anticipated rewards, social expectations, and gender norms [18,19].

Within this theoretical frame, adventure sports tourism differs from conventional sightseeing by foregrounding motives such as skill mastery, testing personal limits, and active immersion in unfamiliar environments [10]. Prior research suggests that participation in high-risk activities is often driven by intrinsic factors such as the pursuit of thrill, personal growth, and self-transcendence, while extrinsic considerations including social recognition, peer comparison, and destination attributes also play an important role [20]. Together, these motives provide the motivational basis for participation intention.

A gendered perspective adds nuance. Compared with men’s emphasis on conquest, competitiveness, or external control, many women place greater weight on emotional depth, pathways to self-development, and the affirmation of identity in social contexts [19]. Qualitative evidence with elite female climbers shows that participation is experienced not only as a physical or technical pursuit but also as an ongoing negotiation of social expectations, in which action carries motives of self-expression and identity work [5]. Although the mix of drivers varies, these patterns are consistent with the self-determination view of autonomous motivation oriented toward autonomy, meaning, and continuing personal development.

In China, this trend also reflects a growing pursuit of self-directed leisure and a negotiation of gendered expectations through physically demanding forms of participation [21,22]. Recent applications of self-determination theory in adventure contexts have primarily linked motivational regulation to downstream psychological outcomes such as memorability and well-being [23]. By contrast, the present study focuses on how intrinsic and extrinsic motivations are translated into participation intention among Chinese women in a collectivist and gendered social context, where institutionalized expectations may complicate the conversion of internal drive into behavioral intention. In this way, the study extends self-determination theory from outcome evaluation to intention formation in adventure sports tourism.

Based on these arguments, the following hypotheses are proposed:

*H1a*: Extrinsic motivation is positively associated with Chinese women’s participation intention in adventure sports tourism.

*H1b*: Intrinsic motivation is positively associated with Chinese women’s participation intention in adventure sports tourism.

### 1.3 The mediating role of women’s empowerment

Empowerment theory, which developed from feminist scholarship and community psychology, provides a useful framework for understanding how individuals strengthen their capacity to act by enhancing self-efficacy, expanding social ties, and engaging with institutions [24,25]. Within the context of adventure sports tourism, this perspective is particularly relevant for women, whose participation is often constrained by gender norms, risk cultures, and structural exclusions from public space [26]. Viewed as a multidimensional process, empowerment helps explain how women translate motivation into participation intention in adventure sports tourism [27].

Women’s empowerment is not a static attribute but a dynamic process involving psychological, social, and political dimensions. Psychological empowerment emphasizes meaning, competence, autonomy, and impact [28]. In adventure settings, it strengthens women’s sense of efficacy and control, thereby supporting the translation of motivation into purposeful participation. In the Chinese context, where traditional family roles and Confucian norms may discourage women’s engagement in high-risk public spaces, psychological empowerment helps participants affirm their capabilities and persist in adventurous pursuits [4,29,30].

Social empowerment highlights the role of interpersonal support, recognition, and community-based legitimacy [31]. In adventure sports tourism, organizations, peer groups, and digital communities can provide women with relational, informational, and symbolic resources that reduce socio-psychological constraints and make participation more socially acceptable [32]. Through these forms of support, social empowerment helps sustain motivational impulses as behavioral intentions under ongoing normative pressure [33].

Political empowerment refers to women’s capacity to gain voice and legitimacy within the institutional arrangements that shape participation opportunities [34]. This dimension extends beyond participation itself by addressing unequal power relations and women’s access to formal influence [35,36]. In China’s sports tourism context, where women have historically had limited institutional visibility, greater involvement in policymaking and organizational decision-making can legitimize their presence in risk-oriented spaces. Political empowerment therefore provides a structural pathway through which motivation is supported not only by personal efficacy or social ties, but also by institutional recognition and stability.

Although the importance of these empowering mechanisms has been widely recognized, existing research across sport and tourism has predominantly framed empowerment as a consequence of participation rather than as a generative process within it [37,38]. Within adventure and leisure contexts, this tendency is reflected in studies that have primarily examined how female participants achieve psychological liberation and transgress traditional bodily boundaries through risk-taking experiences [39]. Nevertheless, these studies predominantly examine empowerment as an isolated retrospective benefit and heavily rely on narratives of individualized resistance. Despite these foundational contributions, there remains a notable scarcity of research treating empowerment as a dynamic proactive mechanism that actively translates initial motivations into sustained behavioral intentions [40]. Furthermore, prior frameworks often isolate specific dimensions of empowerment and thus overlook the complex interplay required to navigate deeply entrenched structural barriers in collectivist societies.

Taken together, these three dimensions suggest that empowerment serves as a multidimensional pathway through which motivation may be translated into participation intention. Based on this reasoning, the study proposes the following hypotheses:

*H2(a,b)*: Psychological empowerment mediates the relationship between adventure motivation and participation intention in adventure sports tourism.

*H2(c,d)*: Social empowerment mediates the relationship between adventure motivation and participation intention in adventure sports tourism.

*H2(e,f)*: Political empowerment mediates the relationship between adventure motivation and participation intention in adventure sports tourism.

### 1.4 The moderating role of risk perception

Adventure sports tourism differs from other forms of leisure travel in that it is shaped by uncertainty, high intensity, and exposure to potentially uncontrollable environments. Unlike broader forms of adventure sports tourism, it emphasizes structured, skill-based participation within standardized safety systems, yet still entails identifiable risks that are only partially controllable [12,14]. These features mean that travelers’ perceptions of risk are central to their decision-making. In tourism research, risk perception is typically defined as the subjective evaluation of uncertainty and the likelihood of negative outcomes, encompassing dimensions such as physical, psychological, social, and financial risks [41,42]. Because such perceptions may differ significantly from objective levels of risk, individual interpretations often become the decisive factor in adventure contexts [43].

The role of risk in this domain is inherently paradoxical. Moderate levels of perceived risk can enhance the sense of excitement and challenge, thus encouraging participation. Conversely, when individuals judge risk to be excessive, fear and avoidance responses can emerge, reducing or even eliminating intention to participate [44,45]. Gendered cultural expectations complicate this further. In many societies, women are socialized to be more sensitive to potential dangers, which may heighten their perception of risk and raise the psychological barriers to entering high-risk environments [10].

Given this complexity, it is reasonable to view risk perception as a boundary condition in the current model. The benefits of empowerment, whether reflected in enhanced competence, stronger social recognition, or institutional support, do not unfold in isolation. Their effectiveness depends on the level of risk women believe they face. When perceived risk is relatively low, empowerment is more likely to strengthen participation intentions. Yet when perceived risk exceeds tolerable limits, its dampening force may outweigh the enabling effects of empowerment, leading even empowered women to withdraw from participation [10,46]. This conditionality suggests that risk perception not only influences the direct pathway from empowerment to intention but also shapes the indirect effect of adventure motivation transmitted through empowerment.

Based on this reasoning, the following hypotheses are proposed:

*H3(a,b,c)*: Risk perception significantly moderates the relationship between women’s empowerment and their participation intention in adventure sports tourism.

*H4*: Risk perception significantly moderates the indirect effect of adventure motivation on participation intention through women’s empowerment.

**Fig 1:**
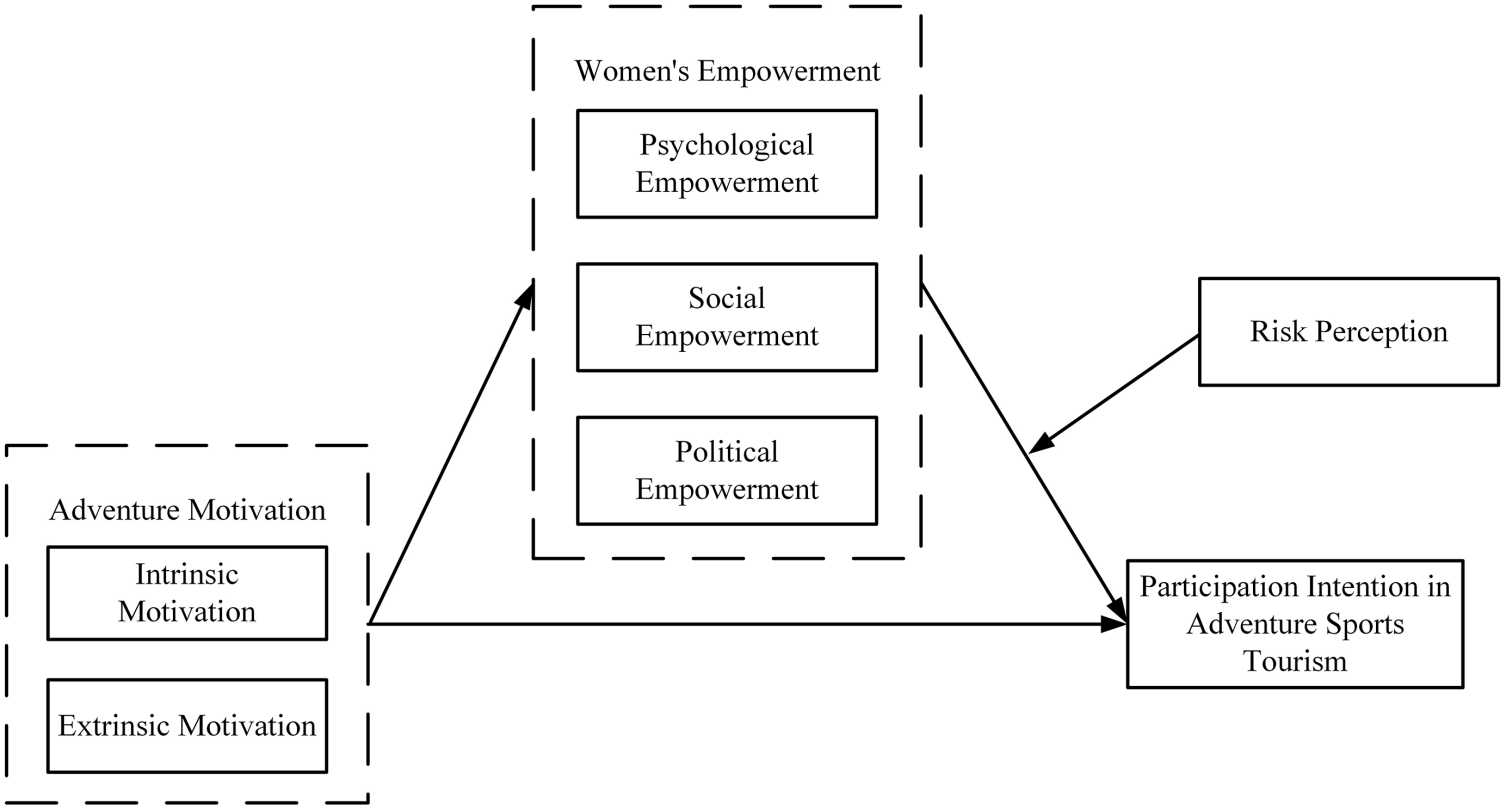
Theoretical model framework.

Accordingly, the theoretical model of this study is illustrated in Fig 1.

## 2. Methods

The study adhered to established ethical principles and was carried out in accordance with recognized guidelines for research involving human participants. For instance, no personally identifiable information was collected. Additionally, a written informed consent form, presented in paper format, was provided to all participants, detailing the study’s purpose and emphasizing the voluntary and confidential nature of participation. The study protocol was reviewed and confirmed exempt by the Ethics Committee of Jiangxi University of Science and Technology.

### 2.1 Research design and participants

This study operated within a post-positivist paradigm, assuming that objective reality is measurable through empirical observation and statistical analysis. Adopting a quantitative deductive approach, the research tested a theoretical model derived from existing literature. Specifically, a cross-sectional survey design was employed to investigate the structural relationships between motivation, empowerment, and behavioral intentions within the target population.

A total of 587 participants were recruited for the survey. After data screening and logical checks, 21 cases were excluded due to invalid responses, resulting in 566 valid participants (Table 1), yielding a valid response rate of 96.42%.

**Table 1:** Sample demographic characteristics (N = 566).

Variable	Category	Frequency (n)	Percentage (%)	Variable	Category	Frequency (n)	Percentage (%)
Age	18–25 years (post-00s)	209	36.9	Education Level	High school or below	75	13.3
	26–35 years (post-90s)	175	30.9		Associate degree	101	17.8
	36–45 years (post-80s)	96	17.0		Bachelor’s degree	198	35.0
	≥45 years (pre-80s)	86	15.2		Postgraduate or above	192	33.9
Mobility Range	Within the province	220	38.9	Average Monthly Income	≤3000 CNY	141	24.9
	Across provinces	226	39.9		3000–5000 CNY	68	12.0
	International travel	120	21.2		5000–8000 CNY	169	29.9
Frequently Participated Adventure Sports	Surfing	199	35.2		≥8000 CNY	188	33.2
	Skiing	192	33.9	Residential Region	Eastern China	215	38.0
	Rock climbing	175	30.9		Central China	192	33.9
Participation Mode	Solo participation	130	23.0		Western China	159	28.1
	With family	85	15.0	Annual Frequency of Adventure Sports Tourism	1–2 times	232	41.0
	With friends	209	36.9		3–5 times	175	30.9
	With colleagues/classmates	142	25.1		≥5 times	159	28.1

Note. CNY = Chinese Yuan.

### 2.2 Instruments

All measures were administered in Chinese using a forward–back translation procedure to ensure conceptual equivalence. Unless otherwise noted, responses were recorded on a five-point Likert scale (1 = strongly disagree to 5 = strongly agree), with higher scores indicating higher levels of the corresponding construct. Reliability and validity statistics, including Cronbach’s α, Composite Reliability (CR), and Average Variance Extracted (AVE), are summarized in Table 2 [47,48].

**Table 2:** Reliability and validity test.

Variables		Components	Cronbach’s α	CR	AVE	$\sqrt{𝐀𝐕𝐄}$
Intrinsic Motivation	To Know	4 items	0.864	0.864	0.615	0.784
	Accomplishment	4 items	0.866	0.867	0.620	0.787
	Experience Stimulation	4 items	0.845	0.846	0.580	0.762
Extrinsic Motivation	Identified Regulation	4 items	0.842	0.846	0.580	0.762
	Introjected Regulation	4 items	0.844	0.846	0.578	0.760
	External Regulation	4 items	0.857	0.857	0.601	0.775
Women’s Empowerment	Psychological Empowerment	5 items	0.849	0.877	0.588	0.767
	Social Empowerment	3 items	0.815	0.817	0.599	0.774
	Political Empowerment	4 items	0.857	0.857	0.600	0.775
Risk Perception		6 items	0.895	0.896	0.589	0.767
Participation Intention		3 items	0.813	0.815	0.596	0.772
χ²/df = 1.188 (<3.0), RMSEA = 0.018 (<0.08), SRMR = 0.028 (<0.08), TLI = 0.986 (>0.95), CFI = 0.987 (>0.95)						

Note. CR = Composite Reliability; AVE = Average Variance Extracted; χ²/df = ratio of chi-square to degrees of freedom; RMSEA = Root Mean Square Error of Approximation; SRMR = Standardized Root Mean Square Residual; TLI = Tucker-Lewis Index; CFI = Comparative Fit Index.

#### 2.2.1 Adventure motivation scale.

Adventure motivation was assessed with an adapted version of the Sport Motivation Scale (SMS) [49] contextualized to the adventure sports tourism setting. Two higher-order dimensions—Intrinsic Motivation and Extrinsic Motivation—were specified, each comprising three first-order components (four items per component; 24 items in total). Intrinsic Motivation consisted of To Know, Accomplishment, and Experience Stimulation, while Extrinsic Motivation included Identified Regulation, Introjected Regulation, and External Regulation. Confirmatory factor analysis (CFA) demonstrated good model fit (χ²/df = 1.269, SRMR = 0.0299, RMSEA = 0.022, GFI = 0.957, AGFI = 0.947, RFI = 0.951, IFI = 0.990, CFI = 0.990). Internal consistency and convergent validity met recommended thresholds, with Cronbach’s α values ranging from 0.842 to 0.866, CR values between 0.846 and 0.867, and AVE values from 0.578 to 0.620 (Table 2).

#### 2.2.2 Women’s empowerment scale.

Women’s empowerment was measured using a multidimensional scale adapted from Boley and McGehee [50] and further supported by subsequent applications in tourism contexts, such as Elshaer et al. [51]. This approach ensured conceptual alignment with the adventure sports tourism setting while acknowledging the broader significance of empowerment in tourism studies. The instrument comprised three first-order dimensions—Psychological Empowerment (five items), Social Empowerment (three items), and Political Empowerment (four items)—for a total of 12 items. CFA indicated an acceptable fit (χ²/df = 1.720, SRMR = 0.0264, RMSEA = 0.036, GFI = 0.975, AGFI = 0.962, RFI = 0.967, IFI = 0.989, CFI = 0.989). Reliability and convergent validity were satisfactory, with Cronbach’s α ranging from 0.815 to 0.857, CR from 0.817 to 0.877, and AVE from 0.588 to 0.600 (Table 2).

#### 2.2.3 Risk perception scale.

Risk perception was evaluated using a six-item scale adapted from Song and Ma [45] to reflect the high-risk characteristics inherent in adventure sports tourism. Items were rated from 1 (strongly disagree) to 5 (strongly agree). CFA results indicated acceptable fit (χ²/df = 2.830, SRMR = 0.0191, RMSEA = 0.057, GFI = 0.984, AGFI = 0.964, RFI = 0.976, IFI = 0.991, CFI = 0.991), and the scale demonstrated strong psychometric properties (α = 0.895, CR = 0.896, AVE = 0.589; Table 2).

#### 2.2.4 Participation intention scale.

Participation intention was measured with three items developed in accordance with the Theory of Planned Behavior [52] to capture respondents’ future intentions to engage in adventure sports tourism. Items were rated on a five-point Likert scale, with higher scores reflecting stronger behavioral intentions. Reliability and convergent validity were adequate (α = 0.813, CR = 0.815, AVE = 0.596; Table 2). Given the three-item unidimensional structure, global CFA fit indices were not emphasized due to known instability with low-indicator models; instead, reliability and AVE were used as primary evidence of measurement quality.

### 2.3 Procedure

To enhance sample representativeness and ecological validity, the study strategically selected three adventure sports tourism destinations in China, each characterized by distinct natural environments: Wanlong Ski Resort in Zhangjiakou (snow-based), Riyue Bay in Wanning (water-based), and Moon Hill in Yangshuo (mountain-based). Rather than arbitrary locations, these sites were selected as the premier, flagship destinations for their respective sports in China. Specifically, Wanlong was widely recognized as a benchmark for commercial skiing [53]; Riyue Bay served as the national surfing training base and the hub of Chinese surf culture [54]; and Moon Hill was globally acknowledged as the cradle of Chinese outdoor rock climbing [55]. Because these iconic hubs attracted enthusiasts from across the country rather than just local residents, sampling at these sites effectively captured a nationally representative tourist base, mitigating the typical geographical limitations of convenience sampling. In contrast, air-based adventure activities, such as skydiving or paragliding, were excluded from this investigation. Unlike in many Western markets, the development of air-based sports in China is constrained by stricter low-altitude airspace controls and high economic barriers to entry. Consequently, the population of participants in air-based sports remains statistically small and less representative of the broader mechanisms driving women’s empowerment in the current Chinese tourism landscape.

Furthermore, a comparison of our sample’s demographic profile with recent national industry data confirmed its representativeness. As detailed in Table 1, the sample predominantly comprises young (18–35 years), highly educated (bachelor’s degree or above) females with middle-to-high income levels. This aligned closely with the demographic consensus from recent national market reports, which identified urban, educated young women as the core demographic driving the contemporary adventure sports tourism market in China [56]. This congruence further supported the ecological validity of the convenience sampling approach employed. The diversity of these environments allowed the investigation to capture behavioral mechanisms across varied physical demands, environmental conditions, risk profiles, and participation patterns.

The survey instrument comprised three sections. To guarantee methodological rigor and sample homogeneity, strict eligibility protocols were established. The inclusion parameters restricted the sample to adult females (aged ≥18) visiting as non-resident tourists and actively engaging in skiing, surfing, or rock climbing. Conversely, exclusion criteria were applied to disqualify males, minors, local residents, venue staff, and passive spectators. The first section included screening questions to validate these eligibility parameters. The second section collected demographic information, including age, education level, income, place of residence, and frequently participated adventure sports. The third section assessed the 45 core psychological items related to the study’s conceptual model. The study adhered to strict ethical guidelines, ensuring anonymity and voluntary participation. All respondents were informed that the data would be used exclusively for academic research and that no personally identifiable information would be collected or disclosed.

To mathematically justify the adequacy of the final sample size (n = 566) for evaluating complex structural relationships, a rigorous statistical power analysis was executed utilizing G*Power 3.1.9.7 [57]. Initially, an *a priori* power analysis for a linear multiple regression (fixed model, R^2^ deviation from zero) was conducted to detect conditional indirect effects. To ensure a conservative estimation, the parameters were set to detect a small-to-medium effect size (f^2^ = 0.05), with a significance criterion of α = 0.05, a target statistical power of 1-β = 0.80, and 9 predictors (accounting for the independent variables, mediators, the moderator, and requisite interaction terms within the moderated mediation framework). The calculation yielded a minimum required sample size of 322 participants, which was substantially surpassed by the empirical sample (n = 566). Moreover, a *post-hoc* power analysis was performed to verify the achieved power. Based on the maximum explained variance observed in the structural estimations (R^2^ = 0.376, yielding an actual effect size of f^2^ = 0.602), the post-hoc statistical power exceeded 0.999. Finally, aligning with empirical simulation studies on bias-corrected bootstrapping, a sample size surpassing 400 provides robust statistical power for reliably detecting multi-path moderated mediation effects [58]. Consequently, the sample size is confirmed to be exceptionally adequate for the hypothesized model.

A convenience sampling strategy was employed, integrating on-site distribution and online dissemination of the questionnaire. The data collection proceeded in two phases. The pilot survey was conducted from January 7 to January 28, 2025, targeting tourists actively engaged in skiing, surfing, and rock climbing. A total of 108 questionnaires were returned, of which 101 were valid (53 online, 48 on-site), yielding a response rate of 93.51%. To verify the psychometric properties and dimensional stability of the adapted scales prior to large-scale administration, an exploratory factor analysis (EFA) and reliability tests were conducted on the pilot data. The data demonstrated excellent suitability for factor extraction, with a Kaiser-Meyer-Olkin (KMO) measure of sampling adequacy at 0.872 and a highly significant Bartlett’s test of sphericity (p < 0.001). The EFA yielded a clear factor structure that explained 71.34% of the cumulative variance, with all measurement items exhibiting robust factor loadings above 0.65 and no problematic cross-loadings. Furthermore, the internal consistency was highly satisfactory, as Cronbach’s α coefficients for all measurement constructs ranged from 0.824 to 0.918, substantially exceeding the recommended 0.70 threshold. Subsequently, the formal survey was carried out from February 2 to April 28, 2025, following the same mixed-method approach. A total of 587 questionnaires were collected, with 21 invalid cases excluded after rigorous data screening and logical checks, resulting in 566 valid responses, achieving a response rate of 96.42%. Crucially, this final sample size substantially exceeds the *a priori* calculated minimum threshold of 322, thereby ensuring highly robust statistical power for the subsequent structural estimations. To ensure data authenticity and validity, the research team implemented stringent quality control measures, including detection of duplicate IP addresses and identification of abnormal response times. Additionally, spot verification with randomly selected participants was conducted to minimize potential sampling bias.

### 2.4 Data analysis

All statistical analyses were conducted using IBM SPSS Statistics 26 (IBM Corp., Armonk, NY, USA) and AMOS 26 (IBM Corp., Armonk, NY, USA) to ensure a comprehensive and rigorous examination of the hypothesized relationships. The analytical procedure proceeded in three sequential stages.

First, given the reliance on self-reported cross-sectional data, a rigorous multi-step diagnostic procedure was employed to assess the potential influence of common method bias (CMB). Initially, Harman’s single-factor test was performed, subjecting all measurement items to an unrotated principal component analysis to determine whether the variance explained by the first major factor exceeded the critical threshold of 40% [59,60]. To address the recognized methodological limitations of relying solely on this traditional approach, two additional robust statistical evaluations were implemented. A full-collinearity assessment was conducted to calculate the Variance Inflation Factors (VIF) for all latent constructs, utilizing a conservative upper limit of 5.0 to detect pathological method collinearity [61]. Finally, an unmeasured latent method construct (ULMC) analysis was executed [60]. This procedure involved introducing an unmeasured common method factor into the baseline confirmatory factor analysis (CFA) model and conducting a nested model comparison to statistically verify whether the inclusion of the method factor significantly altered the underlying measurement structure.

Second, descriptive statistics and Pearson’s correlation analyses were conducted to examine central tendency, variability, and bivariate associations among the study variables, thereby providing an initial overview of the data structure and interrelationships.

Third, the primary hypothesis testing was conducted using Hayes’ PROCESS macro (version 4.2) in SPSS [62]. Mediation analyses were employed to test the indirect effects of women’s empowerment on the relationships between intrinsic and extrinsic motivation and participation intention. To further investigate conditional effects, moderated mediation analyses were performed to examine whether risk perception moderated the path from women’s empowerment to participation intention, as well as the overall indirect effect of adventure motivation. Bias-corrected bootstrapping with 5,000 resamples was used to generate 95% confidence intervals for indirect and interaction effects, with statistical significance determined when the confidence intervals did not include zero [63]. To aid interpretation of moderation, simple slope analyses were conducted to visually depict the direction and magnitude of the interaction effects [64]. All statistical tests were two-tailed with a significance level set at p < 0.05. In accordance with the ordinary least squares regression framework, unstandardized coefficients and their standard errors are primarily reported, with standardized coefficients additionally presented where appropriate to facilitate cross-model comparisons.

## 3. Results

### 3.1. Common method bias test

Given the reliance on self-reported cross-sectional survey data, common method bias could potentially inflate the observed structural relationships. To mitigate this concern, a comprehensive multi-step diagnostic approach was employed in accordance with established methodological guidelines.

First, Harman’s single-factor test was conducted via an unrotated principal component analysis encompassing all measurement items. The results reveal that the first major factor accounts for 32.05% of the total variance. This value falls below the widely accepted 40% threshold, indicating that a single general factor does not emerge to explain the majority of the covariance among the measures.

Second, a full-collinearity assessment was performed by computing the VIF for all latent constructs. The maximum VIF value observed is 1.954. This value satisfies the conservative upper limit of 5.0 and even the stricter criterion of 3.3, suggesting that the data are free from pathological common method collinearity.

Third, an inspection of the inter-construct correlation matrix reveals the absence of uniformly high correlations. Given that no correlation coefficient exceeds the 0.90 threshold, it is improbable that a single method-driven factor dominates the dataset.

Finally, to provide more rigorous statistical evidence, an ULMC analysis was performed in AMOS. Specifically, an unmeasured common method factor was introduced into the baseline measurement model and linked to all observed indicators. A nested model comparison between the baseline CFA model and the ULMC model demonstrates no statistically significant difference in model fit (Δχ² = 7.124, Δdf = 55, p = 1.000 > 0.05). Taken together, this statistical triangulation provides converging evidence that common method variance is unlikely to act as a pervasive confounding factor, thereby supporting the validity and robustness of the structural findings.

### 3.2. Descriptive statistics and correlation analysis

Table 3 presents the descriptive statistics and bivariate correlations for all study variables (N = 566). The mean scores for all scales are above the theoretical midpoint, ranging from 3.44 to 3.61, with standard deviations between 0.697 and 0.890, indicating generally favorable evaluations across the measured constructs. Intrinsic motivation and extrinsic motivation are moderately correlated (r = 0.545, p < 0.01). Both forms of motivation are positively associated with participation intention, with correlations of 0.582 for intrinsic motivation and 0.498 for extrinsic motivation (both ps < .01). Each of the three empowerment dimensions—psychological, social, and political—is positively related to participation intention, with correlation coefficients of 0.440, 0.367, and 0.453, respectively (all ps < .01), and the empowerment dimensions are moderately intercorrelated (ranging from 0.479 to 0.607). Risk perception demonstrates small-to-moderate positive correlations with the other variables, including participation intention (r = 0.272, p < 0.01). None of the correlations exceeds 0.70, suggesting a low likelihood of multicollinearity and supporting the suitability of the data for subsequent multivariate analyses.

**Table 3:** Descriptive statistics and correlations of variable (N = 566).

Variables	Mean	SD	1	2	3	4	5	6	7
1. Intrinsic Motivation	3.457	0.727	1						
2. Extrinsic Motivation	3.548	0.697	0.545**	1					
3. Psychological Empowerment	3.448	0.882	0.534**	0.488**	1				
4. Social Empowerment	3.444	0.889	0.458**	0.385**	0.517**	1			
5. Political Empowerment	3.523	0.890	0.542**	0.476**	0.607**	0.479**	1		
6. Risk Perception	3.552	0.829	0.409**	0.353**	0.404**	0.361**	0.399**	1	
7. Participation Intention	3.611	0.867	0.582**	0.498**	0.440**	0.367**	0.453**	0.272**	1

Note. M = Mean; SD = Standard Deviation; **p<0.01.

### 3.3 Hypothesis test

#### 3.3.1 Structural model adequacy and configural invariance.

Prior to interpreting the specific path coefficients and testing the hypothesized moderated mediation framework, we evaluated the adequacy of the full structural model based on the two-step approach recommended by Anderson and Gerbing [65]. To confirm the structural integrity of the theoretical relationships without the estimation issues often introduced by endogenous interaction terms in covariance-based structural equation modeling, a baseline latent structural model was specified. This model incorporated the latent constructs of all main predictors, mediators, the moderator, and the outcome.

As detailed in Table 4, the structural model showed a good fit to the data. The ratio of chi-square to degrees of freedom (χ²/df) is 1.197, falling significantly below the rigorous threshold of 3.0. The root mean square error of approximation (RMSEA) and the standardized root mean square residual (SRMR) are 0.019 and 0.033, respectively, both well within the recommended upper limit of 0.08. Furthermore, the Tucker-Lewis Index (TLI) and the Comparative Fit Index (CFI) reach 0.985 and 0.986, respectively, substantially exceeding the 0.95 benchmark for superior fit. Taken together, these fit statistics indicate that the hypothesized structural framework was well supported by the observed data, providing an appropriate basis for the subsequent evaluation of specific pathways and conditional moderation effects.

**Table 4:** Structural model fit indices.

Fitting index	χ²/df	RMSEA	SRMR	TLI	CFI
Reference standard	<3.000	<0.08	<0.08	>0.95	>0.95
Overall structural model (n = 566)	1.197	0.019	0.033	0.985	0.986
Surfing (n = 199)	1.195	0.031	0.050	0.957	0.961
Skiing (n = 192)	0.997	0.000	0.040	1.001	1.000
Rock climbing (n = 175)	1.226	0.036	0.049	0.948	0.953

Note. χ²/df = ratio of chi-square to degrees of freedom; RMSEA = Root Mean Square Error of Approximation; SRMR = Standardized Root Mean Square Residual; TLI = Tucker-Lewis Index; CFI = Comparative Fit Index.

Furthermore, to address potential measurement variances across different adventure contexts, we established configural invariance by evaluating the baseline measurement model across the three distinct sports sub-samples (Table 4). The model demonstrates robust fit for the surfing group (χ²/df = 1.195, RMSEA = 0.031, SRMR = 0.050, TLI = 0.957, CFI = 0.961) and adequate fit for the rock climbing group (χ²/df = 1.226, RMSEA = 0.036, SRMR = 0.049, TLI = 0.948, CFI = 0.953). For the skiing sub-sample, the model also converges successfully (χ²/df = 0.997, RMSEA = 0.000, SRMR = 0.040, TLI = 1.001, CFI = 1.000). The exceptional absolute fit indices for the skiing group (e.g., CFI capped at 1.000 and RMSEA truncated at 0.000) are recognized statistical artifacts resulting from the chi-square value being slightly smaller than the degrees of freedom (χ² < df), a common mathematical phenomenon when evaluating highly complex models within partitioned sub-samples. Collectively, these results suggest that female participants across the three adventure sports conceptualized the adapted constructs within a broadly consistent dimensional structure. This configural invariance supports the use of the pooled sample (N = 566) for estimating the overall structural relationships.

#### 3.3.2 Direct effect test.

As presented in Table 5, the direct effect models demonstrate significant positive associations between adventure motivation and participation intention. In Model 1, intrinsic motivation is positively related to participation intention, with an unstandardized coefficient of 0.694 (SE = 0.041, p < 0.001), explaining 33.8% of the variance (R² = .338, F = 288.249, p < 0.001). In Model 2, extrinsic motivation also exhibits a positive association with participation intention, yielding an unstandardized coefficient of 0.620 (SE = 0.045, p < 0.001) and accounting for 24.8% of the variance (R² = .248, F = 186.261, p < 0.001). Although these models were estimated separately, the magnitude of the coefficients suggests that intrinsic motivation is a relatively stronger predictor of participation intention within this sample. These findings provide empirical support for hypotheses H1a and H1b.

**Table 5:** Regression coefficients for paths in the mediation model.

Variable	Participation Intention		Psychological Empowerment		Social Empowerment		Political Empowerment	
**Model 1**	**Model 2**	**Model 3**	**Model 4**	**Model 5**	**Model 6**	**Model 7**	**Model 8**
Constant	1.212***	1.410***	1.210***	1.258***	1.510***	1.703***	1.228***	1.366***
	(−0.144)	(−0.164)	(0.153)	(0.168)	(0.162)	(0.179)	(0.153)	(0.171)
IM	0.694***		0.647***		0.559***		0.664***	
	(−0.041)		(0.043)		(0.046)		(0.043)	
EM		0.620***		0.617***		0.491***		0.608***
		(−0.045)		(0.047)		(0.050)		(0.047)
R^2^	0.338	0.248	0.285	0.238	0.209	0.148	0.294	0.226
F	288.249	186.261	224.535	175.875	149.303	97.990	234.699	165.030
	p < 0.001	p < 0.001	p < 0.001	p < 0.001	p < 0.001	p < 0.001	p < 0.001	p < 0.001

Note. IM = Intrinsic Motivation; EM = Extrinsic Motivation; ***p < 0.001; Values outside parentheses represent unstandardized coefficients, and values in parentheses are standard errors.

#### 3.3.3 Mediating effect test.

Mediation analysis was performed using a bias-corrected bootstrapping procedure with 5,000 resamples, in line with established recommendations for testing indirect effects [66] (Tables 5 and 6). The results indicate that both intrinsic and extrinsic motivation are positively associated with each of the three empowerment dimensions. For intrinsic motivation, the path coefficients range from 0.559 to 0.664, with standard errors between 0.043 and 0.046, and all p-values less than.001. For extrinsic motivation, the coefficients range from 0.491 to 0.617, with standard errors between 0.047 and 0.050, and all p-values less than.001.

**Table 6:** Bias-corrected bootstrapping results for indirect effects.

Effect Type	Paths	Effect	BootSE	BootLLCI	BootULCI	Proportion
Independent Variable: Intrinsic Motivation						
Total Effect	IM → PI	0.694	0.041	0.614	0.774	–
Direct Effect	IM → PI	0.515	0.051	0.415	0.614	74.207%
Total Indirect Effects	IM → WE → PI	0.179	0.037	0.107	0.252	25.793%
Indirect Effect 1	IM → PsE → PI	0.065	0.032	0.005	0.128	9.366%
Indirect Effect 2	IM → SoE → PI	0.029	0.023	−0.015	0.074	4.179%
Indirect Effect 3	IM → PoE → PI	0.085	0.033	0.024	0.153	12.248%
Independent Variable: Extrinsic Motivation						
Total Effect	EM → PI	0.620	0.045	0.531	0.710	–
Direct Effect	EM → PI	0.390	0.051	0.289	0.491	62.903%
Total Indirect Effects	EM → WE → PI	0.230	0.037	0.161	0.307	37.097%
Indirect Effect 1	EM → PsE → PI	0.079	0.032	0.018	0.146	12.742%
Indirect Effect 2	EM → SoE → PI	0.044	0.022	0.004	0.090	7.097%
Indirect Effect 3	EM → PoE → PI	0.107	0.033	0.047	0.173	17.258%

Note. IM = Intrinsic Motivation; EM = Extrinsic Motivation; WE = Women’s Empowerment; PsE = Psychological Empowerment; SoE = Social Empowerment; PoE = Political Empowerment; PI = Participation Intention; BootSE = the bootstrapped standard error; BootLLCI = lower limit of the 95% confidence interval; BootULCI = upper limit of the 95% confidence interval.

Within the intrinsic motivation model, the total indirect effect via empowerment is statistically significant, with an unstandardized coefficient of 0.179 (BootSE = 0.037, 95% CI [0.107, 0.252]), accounting for 25.79% of the total effect. The direct effect remains significant at 0.515 (BootSE = 0.051, 95% CI [0.415, 0.614]), indicating partial mediation. Among the three empowerment pathways, significant indirect effects are observed for psychological empowerment (0.065, 95% CI [0.005, 0.128]) and political empowerment (0.085, 95% CI [0.024, 0.153]), whereas the pathway through social empowerment is not supported (0.029, 95% CI [−0.015, 0.074]).

For extrinsic motivation, the total indirect effect is also statistically significant, with an unstandardized coefficient of 0.230 (BootSE = 0.037, 95% CI [0.161, 0.307]), representing 37.10% of the total effect. The direct path remains significant at 0.390 (BootSE = 0.051, 95% CI [0.289, 0.491]), again suggesting partial mediation. In contrast to intrinsic motivation, all three empowerment pathways reach statistical significance in the extrinsic motivation model. The indirect effect via psychological empowerment is 0.079 (95% CI [0.018, 0.146]), via social empowerment is 0.044 (95% CI [0.004, 0.090]), and via political empowerment is 0.107 (95% CI [0.047, 0.173]), with political empowerment contributing the largest proportion of the indirect effect.

In sum, the findings provide support for hypotheses H2a, H2b, H2d, H2e, and H2f, indicating that empowerment mediates the association between both intrinsic and extrinsic motivation and participation intention across several empowerment dimensions. However, hypothesis H2c is not supported, as social empowerment does not significantly mediate the link between intrinsic motivation and participation intention. These results highlight that while the mediating role of empowerment is evident, the specific pattern of mediation differs across dimensions, with extrinsic motivation displaying a broader and stronger mediation profile.

#### 3.3.4 A moderated mediating effect test.

Table 7 presents the regression results for the moderation and moderated mediation analyses. Models 10–12 test the direct moderating effect of risk perception on the relationship between each empowerment dimension and participation intention. The interaction terms are consistently negative and statistically significant across all three dimensions: psychological empowerment * risk perception (b = −0.182, SE = 0.040, p < 0.001), social empowerment * risk perception (b = −0.203, SE = 0.039, p < 0.001), and political empowerment * risk perception (b = −0.144, SE = 0.039, p < 0.001). These results indicate that higher levels of risk perception attenuate the positive effect of empowerment on participation intention.

**Table 7:** Regression results for moderation and moderated mediation predicting participation intention.

Variable	Participation Intention								
**Model 10**	**Model 11**	**Model 12**	**Model 13**	**Model 14**	**Model 15**	**Model 16**	**Model 17**	**Model 18**
Constant	−0.106	−0.192	0.218	−0.055	−0.767	−0.745	−0.906*	0.196	−0.568
	(0.460)	(0.454)	(0.456)	(0.417)	(0.438)	(0.402)	(0.424)	(0.414)	(0.437)
IM				0.552***		0.601***		0.550***	
				(0.050)		(0.047)		(0.050)	
EM					0.442***		0.487***		0.442***
					(0.051)		(0.049)		(0.050)
PsE	1.010***			0.533***	0.814***				
	(0.142)			(0.136)	(0.136)				
SoE		0.985***				0.685***	0.788***		
		(0.139)				(0.125)	(0.130)		
PoE			0.883***					0.444**	0.733***
			(0.136)					(0.130)	(0.129)
RP	0.709***	0.829***	0.600***	0.335**	0.590***	0.561***	0.647***	0.252	0.515***
	(0.136)	(0.136)	(0.138)	(0.128)	(0.129)	(0.121)	(0.126)	(0.129)	(0.129)
PsE*RP	−0.182***			−0.101**	−0.167***				
	(0.040)			(0.037)	(0.038)				
SoE*RP		−0.203***				−0.166***	−0.178***		
		(0.039)				(0.035)	(0.036)		
PoE*RP			−0.144***					−0.074*	−0.141***
			(0.039)					(0.036)	(0.037)
R^2^	0.232	0.195	0.234	0.370	0.324	0.376	0.316	0.370	0.328
F	56.718	45.298	57.227	82.385	67.353	84.612	64.859	82.319	68.338
	p < 0.001	p < 0.001	p < 0.001	p < 0.001	p < 0.001	p < 0.001	p < 0.001	p < 0.001	p < 0.001

Note. Values are unstandardized coefficients (b) with standard errors in parentheses. Models 10–12 test the moderation of risk perception on the empowerment–participation intention link. Models 13–18 add intrinsic and extrinsic motivation to assess moderated mediation. All models were estimated using ordinary least squares with two-tailed tests. ***p < 0.001, **p < 0.01, *p < 0.05. IM = Intrinsic Motivation; EM = Extrinsic Motivation; PsE = Psychological Empowerment; SoE = Social Empowerment; PoE = Political Empowerment; RP = Risk Perception.

Models 13–18 incorporate empowerment as a mediator between adventure motivation and participation intention, allowing for the assessment of moderated mediation effects. The interaction terms in these models remain negative and significant for all empowerment dimensions—psychological (b = −0.101, SE = 0.037, p < 0.01; b = −0.167, SE = 0.038, p < 0.001), social (b = −0.166, SE = 0.035, p < 0.001; b = −0.178, SE = 0.036, p < 0.001), and political (b = −0.074, SE = 0.036, p < 0.05; b = −0.141, SE = 0.037, p < 0.001). This pattern confirms that risk perception not only weakens the direct empowerment–intention link but also diminishes the strength of the indirect effect of adventure motivation on participation intention via empowerment.

**Fig 2:**
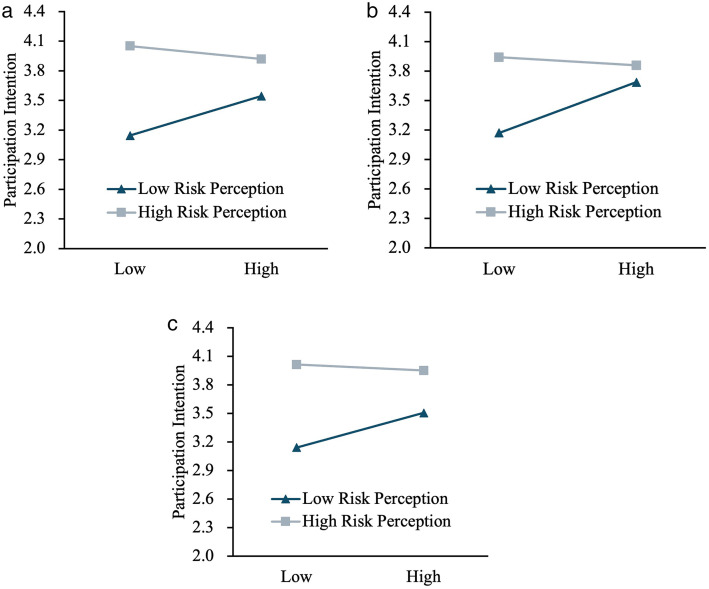
Interactions between empowerment and risk perception on participation intention: (a) Psychological, (b) Social, and (c) Political empowerment. Note. Panels depict simple slopes of empowerment predicting participation intention at low (−1 SD) and high (+1 SD) levels of risk perception. Lines/markers differentiate the two risk-perception levels. All predictors were mean-centered prior to analysis. Simple slopes were computed using Hayes’ PROCESS with 5,000 bias-corrected bootstrap resamples; 95% confidence intervals are reported in Table 8.

Table 8 and Fig 2 further illustrate this attenuation pattern across different levels of risk perception. Regarding the conditional direct effects (Model 1), the simple slope predicting participation intention decreases for psychological empowerment from 0.514 (SE = 0.049; 95% CI [0.419, 0.610]) at one standard deviation below the mean of risk perception, to 0.213 (SE = 0.055; 95% CI [0.104, 0.321]) at one standard deviation above the mean. For social empowerment, the slope sharply declines from 0.433 (SE = 0.047; 95% CI [0.340, 0.525]) to a non-significant 0.097 (SE = 0.056; 95% CI [−0.014, 0.207]) under high risk conditions. Conversely, the slope for political empowerment remains statistically significant across all levels, although it decreases more gradually, from 0.490 to 0.250.

**Table 8:** Conditional direct and indirect effects at specific levels of risk perception.

Mediator Variable	Moderator Variable	Effect	SE	LLCI	ULCI
**Model 1**					
Psychological Empowerment	M-1SD	0.514	0.049	0.419	0.610
	M	0.364	0.040	0.285	0.442
	M + 1SD	0.213	0.055	0.104	0.321
Social Empowerment	M-1SD	0.433	0.047	0.340	0.525
	M	0.265	0.040	0.186	0.344
	M + 1SD	0.097	0.056	−0.014	0.207
Political Empowerment	M-1SD	0.490	0.046	0.399	0.580
	M	0.370	0.040	0.291	0.449
	M + 1SD	0.250	0.056	0.140	0.361
**Model 14 (IM)**					
Psychological Empowerment	M-1SD	0.258	0.050	0.160	0.356
	M	0.174	0.040	0.095	0.253
	M + 1SD	0.090	0.051	−0.011	0.191
Social Empowerment	M-1SD	0.234	0.044	0.147	0.321
	M	0.096	0.038	0.022	0.171
	M + 1SD	−0.041	0.051	−0.140	0.059
Political Empowerment	M-1SD	0.243	0.048	0.150	0.337
	M	0.182	0.040	0.103	0.261
	M + 1SD	0.121	0.052	0.018	0.224
**Model 14 (EM)**					
Psychological Empowerment	M-1SD	0.360	0.049	0.264	0.456
	M	0.222	0.041	0.141	0.302
	M + 1SD	0.084	0.054	−0.022	0.190
Social Empowerment	M-1SD	0.304	0.045	0.215	0.393
	M	0.156	0.039	0.080	0.232
	M + 1SD	0.009	0.053	−0.094	0.112
Political Empowerment	M-1SD	0.349	0.046	0.259	0.440
	M	0.233	0.041	0.153	0.313
	M + 1SD	0.116	0.055	0.009	0.224

Note. For Model 1, “Effect” denotes the conditional direct effect of the specific empowerment dimension predicting participation intention. For Model 14 (IM) and Model 14 (EM), “Effect” denotes the conditional indirect effect of intrinsic motivation (IM) and extrinsic motivation (EM) on participation intention via the respective empowerment dimension. SE = Bootstrapped Standard Error; LLCI = Lower Limit of the 95% Confidence Interval; ULCI = Upper Limit of the 95% Confidence Interval; M = mean level of risk perception; M − 1SD = one standard deviation below the mean; M + 1SD = one standard deviation above the mean.

To further test the moderated mediation framework (H4), we examined the conditional indirect effects using PROCESS Model 14. The results show that the indirect pathways from adventure motivation to participation intention are significantly contingent upon risk perception. For intrinsic motivation (IM), the indirect effect via psychological empowerment weakens from 0.258 (95% CI [0.160, 0.356]) at low risk perception to a non-significant 0.090 (95% CI [−0.011, 0.191]) at high risk perception. A similar attenuation is observed in the social empowerment pathway, dropping from 0.234 (95% CI [0.147, 0.321]) to a non-significant −0.041 (95% CI [−0.140, 0.059]). By contrast, the indirect effect transmitted through political empowerment remains significant across all risk levels, yielding an effect of 0.121 (95% CI [0.018, 0.224]) even when perceived risk is high.

A similar pattern was observed in the extrinsic motivation (EM) model. The conditional indirect effects via psychological and social empowerment both decline to non-significance at high levels of risk perception (Effects = 0.084, 95% CI [−0.022, 0.190] and 0.009, 95% CI [−0.094, 0.112], respectively). However, the political empowerment pathway remains significant, ranging from 0.349 (95% CI [0.259, 0.440]) at low risk to 0.116 (95% CI [0.009, 0.224]) at high risk.

Taken together with the results from Models 13–18, in which intrinsic and extrinsic motivation are included, these findings provide clear support for the presence of moderated mediation. Specifically, the indirect effects from intrinsic and extrinsic motivation to participation intention through empowerment are reduced when risk perception is higher. This indicates that the mediating pathway via empowerment is conditional upon risk perception, consistent with the conceptualization proposed by Preacher, Rucker, and Hayes [67]. The results thereby support both H3, which posits that risk perception moderates the empowerment–participation intention link, and H4, which states that the mediation effect of empowerment is contingent on the level of risk perception.

## 4. Discussion

### 4.1 Motivational predictors of women’s adventure sports tourism participation

This study reveals that intrinsic motivation (b = 0.694, SE = 0.041, p < .001) is more strongly associated with Chinese women’s intentions to participate in adventure sports tourism than extrinsic motivation (b = 0.620, SE = 0.045, p < .001). These findings support the applicability of self-determination theory within the adventure sports tourism context. Prior research highlights that tourists motivated by self-challenge and personal growth are more likely to sustain long-term engagement [68]. Similarly, Chinese women often perceive adventure sports tourism as a pathway for self-expression, identity affirmation, and personal growth, reflecting empowerment processes through travel experiences [6]. This finding resonates with international scholarship on women’s leisure, which posits that adventure serves as a universal space for women to negotiate gendered constraints and reclaim bodily autonomy [18]. However, a subtle distinction exists: while Western narratives often frame this intrinsic drive as a radical “escape” from domesticity, Chinese women appear to view self-growth as a means to construct a modern, cosmopolitan identity that remains compatible with social integration.

Regional research provides further evidence for this interpretation. Luong and Nguyen [69] found that intrinsic motivation, more than extrinsic motivation, predicts sustained participation among Vietnamese female adventure travelers, with risk perception shaping these relationships. Adventure activities provide opportunities to demonstrate resilience, enhance inner strength, and cultivate self-development. In China, the growing awareness of individualism and the pursuit of self-expression among urban women has intensified the role of intrinsic motivation, simultaneously challenging traditional gender roles and reshaping women’s participation in high-risk sports environments [4].

Nevertheless, extrinsic motivation should not be discounted. Social interactions, external recognition, and reputational rewards remain relevant. Munar and Jacobsen [70] demonstrate that sharing experiences through social media fosters engagement by providing external validation. Yoo, Yoon, and Park [71] also emphasize that social support and recognition predict destination choice and tourism behaviors. Here, the cultural specificity of the Chinese context becomes apparent. In collectivist societies such as China, family expectations and social evaluations continue to exert significant influence [33]. Unlike in individualistic cultures where social pressure is often conceptualized primarily as a barrier to leisure, for Chinese women, external validation serves as a crucial mechanism for legitimizing their participation. Women therefore must navigate a “relational self,” negotiating both internal psychological barriers and external pressures from family, peers, and broader social networks [32].

Taken together, this analysis highlights the complex interplay between intrinsic and extrinsic motivations in shaping women’s intentions to engage in adventure sports tourism. Intrinsic motivation appears to provide a more stable foundation, while extrinsic motivation complements it by reinforcing social visibility and normative support. This integrated perspective enriches the understanding of motivational dynamics in Chinese women’s adventure sports tourism participation and underscores the importance of considering both internal and external drivers in program and policy design.

### 4.2 The mediating role of empowerment in the motivation–intention relationship

The mediation results provide further nuance to the relationship between motivation and participation intention by showing how empowerment functions as an intermediary mechanism. For intrinsic motivation, the indirect effect through empowerment accounted for just over a quarter of the total effect (25.79%), with psychological and political empowerment emerging as significant pathways. Social empowerment, however, did not reach statistical significance. By comparison, extrinsic motivation displayed a stronger and broader mediation profile, with 37.10% of the total effect explained through empowerment. In this model, all three empowerment dimensions were significant, and political empowerment in particular carried the largest share of the indirect association. These findings suggest that while both motivational sources are connected to empowerment processes, extrinsic motivation is more consistently linked to the kinds of empowerment that predict participation intentions.

From the perspective of empowerment theory, these results are revealing. Empowerment, as discussed by Kabeer [31] and Zimmerman [29], is not a singular state but a multidimensional process involving psychological strength, social resources, and political voice. The present findings illustrate how these dimensions are differentially activated depending on motivational sources. Intrinsic motivation, which is grounded in self-expression and personal growth, is most strongly connected to psychological empowerment, as it gives women greater confidence and autonomy. This pathway aligns with international research on “embodied empowerment,” which suggests that mastering physical risks allows women globally to challenge the myth of female frailty and cultivate an internal locus of control [72]. Similarly, intrinsic motivation links to political empowerment, which reflects agency in public and often male-dominated domains. The absence of social empowerment in this pathway indicates that self-driven engagement does not necessarily produce wider social validation. Women motivated primarily by internal goals may achieve a sense of autonomy, but such progress may not always be acknowledged within their immediate social circles.

The salience of political empowerment across both models, and especially in the extrinsic motivation pathway, reflects broader features of the Chinese context. This finding offers a distinct contrast to Western feminist leisure scholarship. While international research often frames political empowerment as acts of “resistance” or subversion against established structures [73], our results suggest that for Chinese women, empowerment is more closely tied to acquiring “institutional legitimacy.” In a society shaped by patriarchal traditions and collective values, empowerment is not only about internal confidence but also about claiming visibility and legitimacy in the public sphere. Political empowerment, whether expressed through representation, leadership, or the occupation of social space, is particularly meaningful in this setting as it sanctions women’s presence through formal authority rather than rebellion. For women motivated by external recognition, it is precisely this dimension of empowerment that validates their choices and strengthens their intention to remain engaged in adventure sports.

The muted role of social empowerment in the intrinsic model can also be understood through the dynamics of contemporary Chinese society. This diverges from Western adventure sports tourism literature, which frequently highlights the sufficiency of niche subcultural communities (e.g., climbing tribes) to provide social empowerment independent of mainstream approval. While many urban women in China are increasingly embracing individualistic values, family expectations and social evaluation frameworks remain influential. When participation is driven primarily by internal motives, the lack of visible social recognition may weaken the translation of psychological gains into broader empowerment. Unlike in individualistic cultures where subcultural support often suffices, in the Chinese context, lack of endorsement from the “core” social circle (family and society) creates a legitimacy deficit. Conversely, extrinsic motivation, by its very nature, is closely tied to recognition and validation from others. This makes it more likely to be reinforced through social empowerment, as women draw on familial and peer support to legitimize their engagement in adventure activities.

In sum, these results underline that empowerment mediates the link between motivation and participation intentions in distinctive ways. Intrinsic motivation appears to cultivate depth of psychological strength and autonomy, while extrinsic motivation channels empowerment more effectively through social and political dimensions. For practice, these insights point to the need for multi-layered strategies. Programs that aim to strengthen women’s participation should not only enhance personal efficacy but also create spaces for recognition, visibility, and collective validation. Initiatives such as leadership opportunities within adventure groups, platforms for sharing personal narratives, and events that invite family and community acknowledgment may help bridge individual aspirations with collective endorsement, thereby sustaining women’s engagement in adventure sports tourism.

### 4.3 The moderating influence of risk perception on empowerment and participation intention

The results show that risk perception plays a moderating role in the link between empowerment and participation intention. When levels of perceived risk rise, the positive association between psychological, social, and political empowerment and women’s willingness to engage in adventure sports tourism becomes weaker. The findings also point to a clear pattern: the main effect of risk perception is positive, whereas the interaction effect is negative. In other words, higher risk perception may in general be linked to higher participation, but it simultaneously reduces the strength of the relationship between empowerment and intention. The moderated-mediation analyses further indicate that this weakening extends to the indirect pathways from both intrinsic and extrinsic motivation through empowerment, suggesting that the second stage of the mechanism is strongly shaped by how women assess potential risks [66,67].

Theoretically, this distinct pattern is congruent with the “risk paradox” concept in adventure leisure, where risk functions dialectically as both an intrinsic attraction and a potential deterrent [74,75]. On a general level, higher risk evaluations can be accompanied by stronger desires for challenge and self-expression, reflecting the allure of uncertainty. Yet, when empowerment is expected to transform into concrete behavioral intention, the deterrent aspect of the paradox becomes salient; heightened risk concerns erode its marginal impact, flattening the slope of this relationship. While empowerment provides psychological resources such as competence and control, along with social resources like recognition and support, the accumulation of risk appraisals creates a cognitive load. As attention shifts toward potential hazards, this creates a boundary condition that limits the degree to which empowerment can be translated into actual intention [43].

The results across dimensions also offer important insights. Psychological empowerment continues to promote higher participation intentions even when risk perception is elevated, but its effect weakens. Political empowerment shows relatively more stability, likely because it legitimizes women’s agency in public spaces often dominated by men. By contrast, social empowerment is the most vulnerable: once perceived risks become prominent, external validation from peers or family cannot offset growing concerns, leading to a sharp decline in predictive power.

In the Chinese context, these findings can be understood through the interplay of patriarchal traditions and collectivist orientations. When risk is perceived as low, empowerment aligns with urban women’s growing aspirations for self-development and social belonging, and both psychological and social pathways are more robust. Under heightened risk, however, entrenched cultural narratives of women’s vulnerability and familial responsibility resurface, creating ambivalence within social networks and constraining the translation of empowerment into intention. Political empowerment, by contrast, benefits from symbolic authority and institutional recognition, which can bolster legitimacy and sustain intention even when risk perception is high [4].

For practice, these results suggest that strengthening empowerment alone is insufficient unless paired with strategies that address perceived risks directly. Adventure sports tourism providers may need to implement visible safety protocols, phased skill training, and transparent risk communication to narrow the gap between perceived and actual risks. Peer mentoring systems and group participation structures should be designed to reassure participants rather than reinforce social pressures. Furthermore, giving women formal leadership or safety stewardship roles can enhance political empowerment, which has proven to be the most resilient predictor under high perceived risk. Such efforts may help sustain empowerment’s positive association with participation intention across varying levels of risk perception and mitigate its erosion when concerns about safety become more salient.

Considered as a whole, the findings support H3 and H4 by demonstrating that risk perception moderates the empowerment–intention pathway and conditions the mediating role of empowerment. Political empowerment emerges as the most resilient route under high-risk perception, while social empowerment is the most vulnerable. These associations should be regarded as predictive rather than causal given the cross-sectional design. Future longitudinal or experimental studies could clarify temporal ordering and evaluate whether targeted interventions in risk communication and recognition practices strengthen these conditional pathways.

### 4.4 The Chinese collectivist context: a relational and stratified empowerment

To comprehensively interpret these findings, it is essential to situate the structural relationships within the broader socio-cultural transformations of contemporary China. The empirical anomalies identified in this study include the distinct vulnerability of social empowerment under heightened risk (Fig 2) and the paramount mediating role of political empowerment. These findings diverge significantly from traditional Western paradigms and illuminate the culturally specific mechanics of female empowerment.

In Western leisure scholarship, adventure sports are frequently conceptualized as arenas for radical individual subversion against patriarchal constraints, where peer communities provide sufficient social capital to sustain participation [18]. However, our data suggest that Chinese women navigate a more complex “relational empowerment” [4]. The sharp attenuation of the social empowerment pathway under high risk reflects deep-rooted Confucian familial ethics. In a collectivist framework, engaging in high-risk physical activities often violates the traditional expectations of female bodily preservation and domestic responsibility [76]. Consequently, when objective or perceived risks escalate, informal support from family and immediate social networks rapidly dissipates, rendering social empowerment structurally fragile. To offset this deficit, Chinese women pivot toward institutional legitimacy, conceptualized here as political empowerment. Securing formal representation and discursive space within the adventure community functions not as an act of rebellion, but as a strategic negotiation to justify their participation without entirely rupturing social harmony [4].

Furthermore, the demographic contours of the current sample (Table 1) reveal that this empowerment mechanism is highly stratified. The predominance of highly educated, middle-to-high-income women aged 18–35 underscores that adventure sports tourism in China remains a capital-intensive, urban-centric phenomenon. Under China’s persistent urban-rural dual structure, the pathways to empowerment identified herein are inextricably linked to spatial and economic privileges [77]. For these urban middle-class women, economic capital is converted into physical autonomy. Conversely, for rural or lower-income women confronting fundamental structural and economic constraints, the high-order psychological and political empowerment derived from adventure leisure remains largely inaccessible [33]. Thus, the model primarily captures the empowerment trajectory of a specific, privileged demographic rather than a universal female experience.

Finally, the age distribution points to an underlying generational shift driven by the ongoing “individualization” of Chinese society [78]. For the post-00s cohort (Gen Z), who are digital natives, the strong interplay between extrinsic motivation and empowerment is likely amplified by the pervasive use of social media (e.g., Xiaohongshu), where adventure sports serve as a highly visible currency for digital identity construction [6]. In contrast, for women in their late twenties and thirties, who frequently navigate the intensive “dual burden” of professional advancement and traditional motherhood expectations [33,79], adventure sports tourism may operate primarily as a spatial escape that affords a fleeting yet profound opportunity to reclaim bodily autonomy from entrenched societal surveillance.

In sum, unlike Western models that often equate empowerment with atomized independence, our findings demonstrate that empowerment among Chinese female adventurers is a highly negotiated, stratified, and relational process. It relies on institutional validation to buffer the social vulnerabilities induced by collectivist risk aversion, highlighting a culturally nuanced pathway toward gender equity in leisure.

### 4.5 Limitation and contribution

#### 4.5.1 Limitation and future research.

Despite the rigorous design, several limitations warrant consideration. First, regarding the scope of activities, the sample was drawn from participants in skiing, surfing, and rock climbing. These were strategically selected to represent the three dominant environmental domains of adventure sports tourism in China—snow, water, and mountain terrain. While this choice ensures meaningful coverage of the most mature market segments, it necessitated the exclusion of niche or emerging categories, particularly air-based adventure sports (e.g., skydiving), due to their current low market penetration. Consequently, the findings may not fully generalize to activities with significantly different entry barriers or risk profiles, and future research should expand to these sectors as the market matures.

Second, regarding construct measurement, this study assessed perceived risk as a holistic, unidimensional variable based on an established general scale. Although this approach effectively captured the overall risk thresholds required for the current moderated mediation model, it inherently condenses the multifaceted nature of risk. In the context of adventure sports, risk perception encompasses distinct dimensions, including physical hazards related to bodily safety and socio-psychological vulnerabilities involving social stigma or normative pressures. These distinct facets could theoretically interact with empowerment pathways in divergent ways. For example, physical risk might primarily attenuate psychological empowerment by elevating internal fear, whereas socio-psychological risk could more severely disrupt social empowerment mechanisms within a collectivist environment. Consequently, adopting multidimensional risk perception scales in future research would permit more nuanced sub-scale analyses. Such an approach could clarify whether distinct risk dimensions differentially regulate the motivational mechanisms underlying adventure sports tourism participation.

Third, regarding temporal ordering and methodological constraints, the current reliance on self-reported and cross-sectional data limits the capacity to draw firm causal inferences among motivation, empowerment, risk perception, and participation intention. Although our directional hypotheses are theoretically grounded in self-determination theory and Empowerment Theory, it remains difficult to statistically eliminate the possibility of reverse causality or endogeneity. Specifically, self-reported data are susceptible to omitted variable bias. Unobserved individual differences, such as baseline risk tolerance or physical self-efficacy, may influence both perceived empowerment and participation intention, thereby confounding the observed associations. Furthermore, regarding reverse causality, it is plausible that women who already possess strong behavioral intentions or prior adventure experience may retrospectively report higher levels of empowerment and intrinsic motivation rather than these factors primarily driving their intention. To address these methodological constraints, longitudinal studies tracking the sequential pathways from motivation to empowerment and subsequent participation over time, as well as experimental manipulations of specific empowerment interventions, are strongly recommended for future research to better establish temporal precedence and mitigate endogeneity concerns.

Fourth, the study did not stratify participants by residential location. Given China’s urban-rural dual structure, patriarchal constraints and social resistance to high-risk leisure may manifest differently across settings. As adventure sports tourism is currently a high-consumption activity, the findings likely predominantly reflect the experiences of urban women. Future research could explicitly incorporate the rural-urban variable to examine potential heterogeneity in empowerment pathways and explore whether specific cultural adaptations are required.

Fifth, while this study treated the three empowerment dimensions as independent parallel mediators to isolate their unique contributions and maintain statistical parsimony, they may theoretically exert synergistic effects. To prevent severe multicollinearity within our complex moderated mediation model, we did not explicitly test interactions among these mediators. Future research could beneficially employ configurational approaches (e.g., fsQCA) or latent interaction models to explicitly untangle how these dimensions interactively drive high-risk leisure participation.

Finally, the reliance on on-site convenience sampling, while practical for accessing transient tourist populations, introduces inherent selection and engaged-participant biases. Because the data were collected at high-profile adventure destinations (e.g., Wanlong Ski Resort), the sample inherently comprises women who not only possess greater financial resources but also have already overcome initial structural and psychological barriers to participation. Consequently, the findings primarily reflect the empowerment and risk negotiation mechanisms of highly committed, active tourists, potentially inflating overall estimates of empowerment and participation intention. These results may not fully capture the constraints faced by potential female tourists whose exceptionally high risk perception, lack of systemic empowerment, or economic limitations prevented them from entering the adventure space entirely. To enhance generalizability, future scholarship should adopt broader, population-based probability sampling strategies, such as systematic selection or partnering with national sports associations. Crucially, subsequent studies should include non-participants and drop-outs to compare the absolute barrier thresholds against the active negotiation mechanisms identified in this study.

#### 4.5.2 Contribution and suggestion.

***4.5.2.1 Theoretical contributions.*** This study offers several critical theoretical contributions to the literature on gender, leisure, and adventure sports tourism. First, by integrating self-determination theory and empowerment theory, it elucidates the complex psychosocial mechanisms driving Chinese women’s intentions in adventure sports tourism. This integration extends existing gender-focused perspectives by demonstrating that empowerment is not a uniform, monolithic process, but a multidimensional framework deeply mediated by cultural contexts. Specifically, in a collectivist society such as China, women’s participation reflects a delicate negotiation between internal drives for autonomy and external expectations of validation, thereby offering nuanced insights that enrich cross-cultural debates on relational empowerment.

Second, and perhaps most notably, this study significantly advances existing empowerment-based models by delineating the resilient function of political empowerment under extreme risk conditions. Traditional leisure constraints frameworks generally posit that elevated risk perception precipitously curtails women’s participation [18]. Furthermore, Western-centric models often frame political empowerment primarily as an ideological act of “resistance” [73]. In stark contrast, our moderated mediation analysis reveals that in a highly structured collectivist context, political empowerment functions as a critical “structural buffer.” While the indirect effects of psychological and social empowerment erode to non-significance under high risk perception, political empowerment exhibits remarkable resilience by securing institutional legitimacy. It sustains behavioral intention even when internal efficacy and social resources are overwhelmed by fear, thereby refining psychosocial explanations of women’s engagement in high-risk environments.

Finally, methodologically, the proposed framework demonstrates robust predictive validity. While foundational studies have successfully utilized empowerment scales to evaluate general tourism impacts or resident attitudes (e.g., Boley and McGehee [50]; Elshaer et al. [51]), predicting specific, high-risk behavioral intentions is notoriously complex. Our integrated motivation-empowerment-risk model successfully accounts for a substantial 37.6% of the variance in participation intention (R^2^ = 0.376). Translating to a large effect size (f^2^ = 0.602) according to established statistical benchmarks, this quantitative outcome underscores that building upon traditional direct-effect models by incorporating risk perception as a boundary condition provides a significantly more powerful and nuanced explanation of women’s decision-making in high-stakes adventure sport tourism.

***4.5.2.2 Practical implications.*** From a practical standpoint, the findings underscore the necessity for culturally responsive interventions tailored to the specific socio-cultural dynamics of the Chinese market. To translate these empirical insights into actionable management tools, we have synthesized a specific intervention recommendations matrix (Table 9). This framework categorizes strategic initiatives across the three empowerment dimensions, detailing targeted groups, specific implementing stakeholders (e.g., policymakers, destination managers, and tourism operators), expected outcomes, and empirical measurement metrics to strictly guide industry practice.

**Table 9:** Specific intervention recommendations matrix for female adventure sports tourism.

Empowerment Dimension	Specific Intervention Strategy	Target Group	Expected Outcome	Measurement Method
Psychological	Implement tiered risk-grading protocols and gender-specific progressive skill clinics.	Novice practitioners and cohorts exhibiting elevated initial risk perception.	Attenuation of perceived risk distortion and amplification of self-efficacy in navigating physical challenges.	Pre- and post-intervention risk perception inventories and physical competence scales.
Social	Institutionalize formalized mentorship paradigms pairing veteran female athletes with novices.	First-time entrants and individuals lacking established peer-support networks within the adventure subculture.	Cultivation of in-group belongingness and social validation, effectively mitigating the pressures of informal collectivist scrutiny.	Post-intervention social connectedness psychometrics and longitudinal cohort retention rates.
Political	Mandate female representation in governance roles (e.g., ‘Safety Stewards’) and implement affirmative instructor quotas.	Advanced practitioners and institutional decision-makers (e.g., club managers, policymakers).	Consolidation of institutional legitimacy and structural representation, embedding female agency within the organizational hierarchy.	Quantitative tracking of female leadership ratios and participant assessments of institutional inclusivity.

First, for tourism operators and destination managers, to bolster psychological empowerment and mitigate risk distortion, they should implement tiered risk-grading protocols alongside women-specific progressive skill clinics. Unlike generic instruction, these targeted pedagogical environments facilitate the navigation of physical risks within a psychologically secure framework, thereby recalibrating the dissonance between perceived and objective danger. As outlined in Table 9, these initiatives should primarily target novice practitioners or cohorts exhibiting elevated initial risk perception. The efficacy of these psychological interventions can be rigorously evaluated through pre- and post-intervention risk perception inventories and physical competence scales.

Second, regarding social empowerment, destination managers and tourism operators are encouraged to institutionalize formalized mentorship structures wherein veteran female practitioners guide novices. This strategy formalizes peer support networks, providing validation that bolsters confidence without exerting the undue pressure often associated with informal collectivist scrutiny. To ensure the sustained cultivation of in-group belongingness, tourism operators should systematically monitor these target groups using post-intervention social connectedness psychometrics and longitudinal cohort retention rates (Table 9).

Third, specifically for policymakers, political empowerment requires structural reinforcement through the creation of explicit industry-wide pathways for women to assume governance or safety-critical roles. At the operational level, the appointment of women as official ‘Safety Stewards’ or the implementation of affirmative instructor quotas by club managers serves to symbolically and structurally legitimize female agency within these traditionally male-dominated enclaves. The success of embedding female agency within the organizational hierarchy can be quantitatively measured by tracking female leadership ratios and collecting participant assessments regarding institutional inclusivity.

While such measures cannot eliminate risk perception entirely, these targeted structural interventions provide the scaffolding necessary for women to manage safety concerns effectively, thereby fostering sustained engagement in adventure sports tourism. Beyond the Chinese context, these approaches offer transferable frameworks for advancing gender equity in other collectivist or transitional societies where cultural norms continue to mediate women’s participation in high-risk leisure.

## 5. Conclusion

This study shows that both intrinsic and extrinsic motivations are positively related to Chinese women’s intentions to engage in adventure sports tourism, and that empowerment provides an important channel through which these motivations are expressed. Within this pathway, psychological empowerment and political empowerment emerge as relatively stable predictors of participation, while social empowerment appears more contingent on contextual conditions. The results further suggest that when risk perception increases, the positive association between empowerment and intention becomes weaker, reflecting the tension between women’s aspirations for growth and their concerns about safety.

Contextualizing these findings within a collectivist framework offers a critical refinement to empowerment theory in tourism scholarship. Diverging from Western paradigms that typically conceptualize political empowerment as an act of resistance, this study posits that, in the Chinese context, it functions as a mechanism for securing institutional legitimacy. This theoretical distinction highlights that women’s engagement in high-risk leisure is predicated not merely on internal agency, but on the authorized negotiation of public space. Consequently, the research establishes a culturally situated framework for interpreting empowerment, demonstrating how individual aspirations are sustained through structural validation in transitional societies.

## Supporting information

S1 File Adventure motivation scale. This document details the measurement items for intrinsic and extrinsic motivation used in the study.
(DOCX)

S2 File Raw data. This Excel spreadsheet contains the anonymized raw dataset. (XLSX)

S3 File Item-by-item rationale for the adapted empowerment scale. (DOCX)

## References

[1] Cater CI. Playing with risk? Participant perceptions of risk and management implications in adventure tourism. Tour Manag. 2006;27(2):317–25. doi: 10.1016/j.tourman.2004.10.005

[2] Escape Adventures. Travel Trends report 2025: the rise of the female adventurer. 2025. Available from: https://escapeadventures.com/news-a-press/press/travel-trends-report-2025-the-rise-of-the-female-adventurer/

[3] Gardiner S, Janowski I, Kwek A. Self-identity and adventure tourism: cross-country comparisons of youth consumers. Tour Manag Perspect. 2023;46:101061. doi: 10.1016/j.tmp.2022.101061

[4] Yang Y, Siu TW. Mind–body harmonization: women’s sports participation and social transformation in modern china. J Chin Sociol. 2025;12(1):5. doi: 10.1186/s40711-025-00231-5

[5] Galiakbarov Y, Mazbayev O, Onayeva B, Bolatova B, Filimonau V, Sezerel H. Conquering heights, challenging norms: the motives and experiences of elite female climbers in a patriarchal society. J Sustain Tour. 2025:1–26. doi: 10.1080/09669582.2025.2487677

[6] Wang Z, Huang X. Empowerment through solo travel: the self-identity transformation of Chinese women in emerging adulthood. Leis Sci. 2025;:1–22. doi: 10.1080/01490400.2025.2512110

[7] Xinhua N. Outdoor sports emerge as a booming industry in China. Available from: http://www.news.cn/fashion/20240327/34631d084bdb49edaed01e97056bb0a5/c.html. 2024.

[8] Apollo M, Mostowska J, Legut A, Maciuk K, Timothy DJ. Gender differences in competitive adventure sports tourism. J Outdoor Recreat Tour. 2023;42:100604. doi: 10.1016/j.jort.2022.100604

[9] Pawid HGB. Motivation and effects of adventure sports tourism in the Cordillera Administrative Region, Philippines. Connexion. 2023;12(2):74–89.

[10] Pan J, Li H. Motivation, emotion, and intention in adventure sports tourism from a risk margin perspective. J Shanghai Univ Sport. 2020;44:34–42.

[11] Kerr JH, Houge Mackenzie S. Multiple motives for participating in adventure sports. Psychol Sport Exerc. 2012;13(5):649–57. doi: 10.1016/j.psychsport.2012.04.002

[12] Nazari Heris M, Faraji R, Bashiri M. Identifying the development strategies of recreational and adventure sports tourism in Iran with the SOAR approach. Sport Manag J. 2024;16:14–21. doi: 10.22059/jsm.2023.353363.3089

[13] Houge Mackenzie S, Hodge K, Filep S. How does adventure sport tourism enhance well-being? A conceptual model. Tour Recreat Res. 2023;48(1):3–16. doi: 10.1080/02508281.2021.1894043

[14] Karimi J, Soltanian L, Bejani A. Designing the model of the development of adventure sports tourism: grounded theory. Sport Manag Stud. 2020;12:61–82. doi: 10.22089/smrj.2019.6327.2288

[15] Deci EL, Ryan RM. Intrinsic motivation and self-determination in human behavior. Boston, MA: Springer US; 1985.

[16] Ryan RM, Deci EL. Self-determination theory and the facilitation of intrinsic motivation, social development, and well-being. Am Psychol. 2000;55(1):68–78. doi: 10.1037//0003-066x.55.1.68 11392867

[17] Deci EL, Ryan RM. Facilitating optimal motivation and psychological well-being across life’s domains. Can Psychol. 2008;49(1):14–23. doi: 10.1037/0708-5591.49.1.14

[18] Little DE. Women and Adventure recreation: reconstructing leisure constraints and adventure experiences to negotiate continuing participation. J Leis Res. 2002;34(2):157–77. doi: 10.1080/00222216.2002.11949967

[19] Clarke JF, Previte J, Chien PM. Adventurous femininities: the value of adventure for women travelers. J Vacat Mark. 2022;28(2):171–87. doi: 10.1177/13567667211038952

[20] Immonen T, Brymer E, Orth D, Davids K, Feletti F, Liukkonen J, et al. Understanding action and adventure sports participation-an ecological dynamics perspective. Sports Med Open. 2017;3(1):18. doi: 10.1186/s40798-017-0084-1 28447331 PMC5406377

[21] Tang B. Multiple OTAs release data reports: women travelers increasingly spend for “self-pleasure” consumption. China Tour News. 2025.

[22] Yu Z, Gu P. Self-awareness awakening and subject construction of female bicycle tourists. Tour Trib. 2022;37:106–20. doi: 10.19765/j.cnki.1002-5006.2022.06.012

[23] Sahoo D, Nukhu R, Dash S, Chauhan V. Adventure tourism and visitors motivations: a mixed-method study on memorability and psychological well-being. J Hosp Tour Insights. 2026;9: 1385–404. doi: 10.1108/JHTI-04-2025-0466

[24] Zimmerman MA. Psychological empowerment: issues and illustrations. Am J Community Psychol. 1995;23(5):581–99. doi: 10.1007/BF02506983 8851341

[25] Cornwall A, Rivas A-M. From ‘gender equality and ‘women’s empowerment’ to global justice: reclaiming a transformative agenda for gender and development. Third World Q. 2015;36(2):396–415. doi: 10.1080/01436597.2015.1013341

[26] Little DE, Wilson E. Adventure and the gender gap: acknowledging diversity of experience. Loisir Soc. 2005;28(1):185–208. doi: 10.1080/07053436.2005.10707676

[27] Fendt LS, Wilson E. ‘I just push through the barriers because I live for surfing’: how women negotiate their constraints to surf tourism. Ann Leis Res. 2012;15(1):4–18. doi: 10.1080/11745398.2012.670960

[28] Spreitzer GM. Psychological empowerment in the workplace: dimensions, measurement, and validation. Acad Manage J. 1995;38(5):1442–65. doi: 10.5465/256865

[29] Zimmerman MA. Empowerment theory. In: Handbook of Community Psychology. Springer US; 2000. p. 43–63. doi: 10.1007/978-1-4615-4193-6_2

[30] Alsop R, Bertelsen M, Holland J. Empowerment in practice: from analysis to implementation. Washington, DC: World Bank; 2006.

[31] Kabeer N. Resources, agency, achievements: reflections on the measurement of women’s empowerment. Dev Change. 1999;30(3):435–64. doi: 10.1111/1467-7660.00125

[32] Gray T, Norton C, Breault-Hood J, Christie B, Taylor N. Curating a public self: exploring social media images of women in the outdoors. JOREL. 2018;10(2):153–70. doi: 10.18666/jorel-2018-v10-i2-8191

[33] Sheng L, Weirong R. “Family-bound” or “Pulled by Work”?—a study of Chinese women’s social participation and the factors influencing it. Soc Sci China. 2020;41(1):133–58. doi: 10.1080/02529203.2020.1719742

[34] Scheyvens R. Ecotourism and the empowerment of local communities. Tour Manag. 1999;20(2):245–9. doi: 10.1016/S0261-5177(98)00069-7

[35] Rowlands J. Questioning empowerment: working with women in Honduras. Oxford: Oxfam; 1997.

[36] Cornwall A, Edwards J. Introduction: negotiating empowerment. IDS Bull. 2010;41(2):1–9. doi: 10.1111/j.1759-5436.2010.00117.x

[37] Vujko A, Karabašević D, Cvijanović D, Vukotić S, Mirčetić V, Brzaković P. Women’s Empowerment in rural tourism as key to sustainable communities’ transformation. Sustainability. 2024;16(23):10412. doi: 10.3390/su162310412

[38] Guo Q, Yang X, Chen H. The influence of women’s empowerment on tourism involvement and sustainable tourism development: the moderating role of tourism cooperatives. Asia Pac J Tour Res. 2023;28(10):1130–46. doi: 10.1080/10941665.2023.2289401

[39] El-Sisi SA-W, Elzek Y, Soliman M, Al-Romeedy BS. Behavioural motivators of solo female travel in non-western contexts: empirical evidence. J Hosp Tour Insights. 2025;9(3):1236–55. doi: 10.1108/jhti-05-2025-0611

[40] Robina-Ramírez R, Leal-Solís A. Women’s sports tourism as a tool for empowerment and continued participation in sports. Retos. 2026;80:13–30. doi: 10.47197/retos.v80.118803

[41] Cui F, Liu Y, Chang Y, Duan J, Li J. An overview of tourism risk perception. Nat Hazard. 2016;82(1):643–58. doi: 10.1007/s11069-016-2208-1

[42] Chen Y, Cui W. Impact of risk perception on surfers’ intention to revisit is mediated by emotion and experience quality. Trop Geogr. 2023;43:2024–34.

[43] Slovic P. Perception of risk. In: The perception of risk. Routledge; 2000.

[44] Quintal VA, Lee JA, Soutar GN. Risk, uncertainty and the theory of planned behavior: a tourism example. Tour Manag. 2010;31(6):797–805. doi: 10.1016/j.tourman.2009.08.006

[45] Song H, Ma T. The influence of alpine adventure destination image on behavioral intention of sports tourists: the mediating role of risk perception and emotion. J Shandong Sport Univ. 2024;40:116–26.

[46] Ross I. Perceived risk and consumer behavior: a critical review. Adv Consum Res. 1975;2:1–20.

[47] Diamantopoulos A, Siguaw JA. Introducing LISREL: a guide for the uninitiated. London: Sage; 2000.

[48] Fornell C, Larcker DF. Evaluating structural equation models with unobservable variables and measurement error. J Mark Res. 1981;18(1):39–50.

[49] Pelletier LG, Tuson KM, Fortier MS, Vallerand RJ, Briére NM, Blais MR. Toward a New measure of intrinsic motivation, extrinsic motivation, and amotivation in sports: The Sport Motivation Scale (SMS). J Sport Exerc Psychol. 1995;17(1):35–53. doi: 10.1123/jsep.17.1.35

[50] Boley BB, McGehee NG. Measuring empowerment: developing and validating the Resident Empowerment through Tourism Scale (RETS). Tour Manag. 2014;45:85–94. doi: 10.1016/j.tourman.2014.04.003

[51] Elshaer I, Moustafa M, Sobaih AE, Aliedan M, Azazz AMS. The impact of women’s empowerment on sustainable tourism development: mediating role of tourism involvement. Tour Manag Perspect. 2021;38:100815. doi: 10.1016/j.tmp.2021.100815

[52] Ajzen I. The theory of planned behavior. Organ Behav Hum Decis Process. 1991;50(2):179–211. doi: 10.1016/0749-5978(91)90020-T

[53] Wu B. China ski industry white paper (2022–2023). Beijing: ISPO China; 2023.

[54] General Administration of Sport of China. Integration of sports and tourism enhances urban soft power: Hainan water sports season. Available from: https://www.sport.gov.cn/n20001280/n20745751/n20767239/c21434937/content.html. 2020.

[55] People’s Daily. From sightseeing to adventure: S China’s Guilin grows into world-class tourist city amid outdoor tourism boom. Available from: https://en.people.cn/n3/2025/0709/c98649-20337860.html. 2025.

[56] Xinhua News. Playing out a trillion-level industry: China’s outdoor sports market. Available from: http://www.news.cn/sports/20231212/d493f11a0b2d4cb5bc213e5e6737aa4e/c.html. 2023.

[57] Faul F, Erdfelder E, Buchner A, Lang A-G. Statistical power analyses using G*Power 3.1: tests for correlation and regression analyses. Behav Res Methods. 2009;41(4):1149–60. doi: 10.3758/BRM.41.4.1149 19897823

[58] Fritz MS, Mackinnon DP. Required sample size to detect the mediated effect. Psychol Sci. 2007;18(3):233–9. doi: 10.1111/j.1467-9280.2007.01882.x 17444920 PMC2843527

[59] Aguirre-Urreta MI, Hu J. Detecting common method bias: performance of the harman’s single-factor test. ACM SIGMIS Database: DATABASE Adv Inf Syst. 2019;50(2):45–70. doi: 10.1145/3330472.3330477

[60] Podsakoff PM, MacKenzie SB, Lee J-Y, Podsakoff NP. Common method biases in behavioral research: a critical review of the literature and recommended remedies. J Appl Psychol. 2003;88(5):879–903. doi: 10.1037/0021-9010.88.5.879 14516251

[61] Kock N. Common method bias in PLS-SEM: a full collinearity assessment approach. Int J e-Collab. 2015;11:1–10. doi: 10.4018/ijec.2015100101

[62] Hayes AF. Introduction to mediation, moderation, and conditional process analysis. 2nd ed. New York: Guilford Publications; 2017.

[63] Baron RM, Kenny DA. The moderator-mediator variable distinction in social psychological research: conceptual, strategic, and statistical considerations. J Pers Soc Psychol. 1986;51(6):1173–82. doi: 10.1037//0022-3514.51.6.1173 3806354

[64] Hayes AF, Matthes J. Computational procedures for probing interactions in OLS and logistic regression: SPSS and SAS implementations. Behav Res Methods. 2009;41(3):924–36. doi: 10.3758/BRM.41.3.924 19587209

[65] Anderson JC, Gerbing DW. Structural equation modeling in practice: a review and recommended two-step approach. Psychol Bull. 1988;103(3):411–23. doi: 10.1037/0033-2909.103.3.411

[66] Preacher KJ, Hayes AF. Asymptotic and resampling strategies for assessing and comparing indirect effects in multiple mediator models. Behav Res Methods. 2008;40(3):879–91. doi: 10.3758/brm.40.3.879 18697684

[67] Preacher KJ, Rucker DD, Hayes AF. Addressing moderated mediation hypotheses: theory, methods, and prescriptions. Multivariate Behav Res. 2007;42(1):185–227. doi: 10.1080/00273170701341316 26821081

[68] Pomfret G, Bramwell B. The characteristics and motivational decisions of outdoor adventure tourists: a review and analysis. Current Issues in Tourism. 2014;19(14):1447–78. doi: 10.1080/13683500.2014.925430

[69] Luong T-B, Nguyen DTA. The Moderating role of risk perception in the relationships between motivation, attitude, and involvement in adventure activities: a study from young Vietnamese travelers. J Qual Assur Hosp Tour. 2024;27(2):371–95. doi: 10.1080/1528008x.2024.2338779

[70] Munar AM, Jacobsen JK r S. Motivations for sharing tourism experiences through social media. Tour Manag. 2014;43:46–54. doi: 10.1016/j.tourman.2014.01.012

[71] Yoo CK, Yoon D, Park E. Tourist motivation: an integral approach to destination choices. Tour Rev. 2018;73(2):169–85. doi: 10.1108/tr-04-2017-0085

[72] Theberge N. Sport and women’s empowerment. Women’s Studies International Forum. 1987;10(4):387–93. doi: 10.1016/0277-5395(87)90056-2

[73] Shaw SM. Conceptualizing resistance: Women’s leisure as political practice. J Leis Res. 2001;33(2):186–201. doi: 10.1080/00222216.2001.11949937

[74] Gilbertson K, Ewert A. Stability of motivations and risk attractiveness: the adventure recreation experience. Risk Manag. 2015;17(4):276–97. doi: 10.1057/rm.2015.16

[75] Buckley R. Rush as a key motivation in skilled adventure tourism: resolving the risk recreation paradox. Tour Manag. 2012;33(4):961–70. doi: 10.1016/j.tourman.2011.10.002

[76] Raymo JM, Park H, Xie Y, Yeung W-JJ. Marriage and family in east asia: continuity and change. Annu Rev Sociol. 2015;41:471–92. doi: 10.1146/annurev-soc-073014-112428 30078932 PMC6070151

[77] Dong E, Chick G. Leisure constraints in six Chinese cities. Leis Sci. 2012;34:417–35. doi: 10.1080/01490400.2012.714702

[78] Yan Y. The individualization of Chinese society. Oxford: Berg; 2009.

[79] Ji Y. Between tradition and modernity: “Leftover” women in Shanghai. J Marriage Fam. 2015;77(5):1057–73. doi: 10.1111/jomf.12220

