# Supplementary material for: Reframing participation through empowerment: Mechanisms driving Chinese women’s intentions in adventure sports tourism
Source: PLoS One. 2026 May 12;21(5):e0339943. doi: 10.1371/journal.pone.0339943 (PMC13166955; doi:10.1371/journal.pone.0339943)
Supplement: S1 File — This document details the measurement items for intrinsic and extrinsic motivation used in the study. (DOCX) [file pone.0339943.s001.docx]

**S1 File. Adventure Motivation Scale.**

| **Latent Variable** | **Component** | **Item Content** |
| --- | --- | --- |
| **Intrinsic Motivation** | **To Know** | For the pleasure of knowing more about this adventure sports tourism activity. |
|  |  | For the pleasure of discovering new training techniques for this activity. |
|  |  | For the pleasure of learning training techniques I have never tried before. |
|  |  | For the pleasure of discovering new strategies and tactics in this activity. |
|  | **Accomplishment** | For the satisfaction I experience while mastering difficult training techniques. |
|  |  | For the pleasure I feel while improving my weak points in this activity. |
|  |  | For the satisfaction I get from improving my abilities. |
|  |  | For the pleasure I feel when executing high-difficulty movements. |
|  | **Experience Stimulation** | For the excitement and stimulation I feel while participating. |
|  |  | For the excitement I feel when I am deeply involved in the activity. |
|  |  | For the intense emotional experiences I have while doing this activity. |
|  |  | For the feeling of being totally immersed in the activity. |
| **Extrinsic Motivation** | **Identified Regulation** | For the things I learn in this activity that are useful in other areas of my life. |
|  |  | To maintain a healthy body through participation. |
|  |  | For the benefits this activity contributes to my personal development. |
|  |  | To gain the health benefits that are valued by those around me. |
|  | **Introjected Regulation** | To feel good about myself when I engage in this activity. |
|  |  | I would feel bad if I did not spend time doing this activity. |
|  |  | I would feel uncomfortable if I could not participate regularly. |
|  |  | To show others that I am excellent at this activity. |
|  | **External Regulation** | To maintain good relationships with friends through this activity. |
|  |  | To gain recognition from people I know. |
|  |  | To use this activity as a way to interact with others. |
|  |  | To gain glory or other rewards I desire. |
