# Supplementary material for: Reframing participation through empowerment: Mechanisms driving Chinese women’s intentions in adventure sports tourism
Source: PLoS One. 2026 May 12;21(5):e0339943. doi: 10.1371/journal.pone.0339943 (PMC13166955; doi:10.1371/journal.pone.0339943)
Supplement: S3 File — (DOCX) [file pone.0339943.s003.docx]

**S3 File. Item-by-item rationale for the adapted empowerment scale.**

| **Dimension** | **Original Items (Boley & McGehee, 2014)** | **Adapted Items for this study** | **Adaptation Rationale** |
| --- | --- | --- | --- |
| **Psychological** | Tourism in Floyd/Franklin/Botetourt County makes me proud to be a Floyd/Franklin/Botetourt County resident. | Adventure sports tourism makes me proud to be a female adventure sports participant. | Shifted focus from *geographic resident pride* to *gender/identity pride* achieved through adventure sports. |
|  | Tourism in Floyd/Franklin/Botetourt County makes me feel special because people travel to see my county's unique features. | Adventure sports tourism makes me feel special because I can accomplish things many think women cannot do. | Adapted the concept of "uniqueness" from *destination features* to *personal capabilities* challenging traditional gender roles. |
|  | Tourism in Floyd/Franklin/Botetourt County makes me want to tell others about what we have to offer in Floyd/Franklin/Botetourt County. | Adventure sports tourism makes me want to recommend this destination to others. | Maintained the behavioral intention to promote a location, but shifted the subject from *residents promoting their hometown* to *empowered female tourists recommending an adventure destination*. |
|  | Tourism in Floyd/Franklin/Botetourt County reminds me that I have a unique culture to share with visitors. | Adventure sports tourism reminds me that I have a unique story to share with others. | Replaced "unique local culture/visitors" with "unique personal story/others." |
|  | Tourism in Floyd/Franklin/Botetourt County makes me want to work to keep Floyd/Franklin/Botetourt County special. | Adventure sports tourism makes me want to work to keep my identity in this sport strong. | Shifted the commitment from *preserving a destination* to *maintaining an empowering identity in the sport*. |
| **Social** | Tourism in Floyd/Franklin/Botetourt County makes me feel more connected to my community. | Adventure sports tourism makes me feel more connected to my community of female adventurers. | Re-conceptualized "community" from a *local geographic area* to a *gender-based supportive network*. |
|  | Tourism in Floyd/Franklin/Botetourt County fosters a sense of 'community spirit' within me. | Adventure sports tourism fosters a sense of 'community spirit' among female participants within me. | Specified the "community spirit" to the *female adventure subculture*. |
|  | Tourism in Floyd/Franklin/Botetourt County provides ways for me to get involved in my community. | Adventure sports tourism provides ways for me to get involved in the female adventure community. | Adapted "involvement" to mean integrating into *female-centric sporting groups*. |
| **Political** | I feel like I have a voice in Floyd/Franklin/Botetourt County tourism development decisions. | I feel like I have a voice in the adventure sports tourism community. | Adapted from *civic tourism planning* to having *discursive space* in a male-dominated sporting arena. |
|  | I feel like I have access to the decision making process when it comes to tourism in Floyd/Franklin/Botetourt County. | I feel like I have access to the decision making process when it comes to women's participation in adventure. | Maintained exact syntactic structure, shifting the domain from *local tourism policy* to *women's involvement in adventure sports*. |
|  | I feel like my vote makes a difference in how tourism is developed in Floyd/Franklin/Botetourt Co. | I feel like my actions make a difference in how female adventure tourism is viewed in society. | Replaced the literal "vote" with "actions/choices," acting as a catalyst for societal change regarding women's adventure behaviors. |
|  | I feel like I have an outlet to share my concerns about tourism development in Floyd/Franklin/Botetourt Co. | I feel like I have an outlet to share my concerns about women's true needs in adventure sports tourism. | Maintained the "outlet for concerns," focusing specifically on the structural/safety *needs of female participants*. |
